# Host mitochondrial transcriptome response to SARS-CoV-2 in multiple cell models and clinical samples

**DOI:** 10.1038/s41598-020-79552-z

**Published:** 2021-01-08

**Authors:** Brendan Miller, Ana Silverstein, Melanie Flores, Kevin Cao, Hiroshi Kumagai, Hemal H. Mehta, Kelvin Yen, Su- Jeong Kim, Pinchas Cohen

**Affiliations:** 1 Leonard Davis School of Gerontology, University of Southern California, 3715 McClintock Ave, Los Angeles, CA 90089 USA; 2 Graduate School of Health and Sports Science, Juntendo University, Chiba, Japan

**Keywords:** Infection, Gene expression

## Abstract

SARS-CoV-2 induces a muted innate immune response compared to other respiratory viruses. Mitochondrial dynamics might partially mediate this effect of SARS-CoV-2 on innate immunity. Polypeptides encoded by open reading frames of SARS-CoV and SARS-CoV-2 have been shown to localize to mitochondria and disrupt Mitochondrial Antiviral Signaling (MAVS) protein signaling. Therefore, we hypothesized that SARS-CoV-2 would distinctly regulate the mitochondrial transcriptome. We analyzed multiple publicly available RNASeq data derived from primary cells, cell lines, and clinical samples (i.e., BALF and lung). We report that SARS-CoV-2 did not dramatically regulate (1) mtDNA-encoded gene expression or (2) MAVS expression, and (3) SARS-CoV-2 downregulated nuclear-encoded mitochondrial (NEM) genes related to cellular respiration and Complex I.

## Introduction

The emergence of SARS-CoV-2 (COVID19) has stressed global economic and health systems^1^. Prior reports showed that SARS-CoV-2 induced a distinct, yet mild innate immune response that was much lower compared to other respiratory viruses^2^. SARS-CoV-2 infection specifically induced low IFN-I and IFN-III levels across multiple cellular models. This specific attenuated innate immune response could explain how older individuals, who are likely to present age-related immune cell decline, are at risk for SARS-CoV-2 mortality. However, the mechanisms by which SARS-CoV-2 evades innate immune signaling are unclear. One potential mechanism is mitochondrial mediation, as SARS-CoV-2 transcriptomic data were enriched for mitochondrial organization processes^3^. Still, the role of mitochondria in SARS-CoV-2 infection is largely undetermined.

Mitochondria regulate innate immune signaling upon viral infection^4^. Host cell innate immunity is regulated by the Mitochondrial Antiviral Signaling protein (MAVS)^5, 6^. MAVS normally interacts with MFN2 under resting conditions^7^. But after viral infection, mitochondria-associated ER membranes and nearby mitochondria become tethered by MFN2 and RIG-1, forming a complex that recruits TRIM25 and the molecular chaperone 14-3-3e into a translocon structure^8^. This translocon localizes to the mitochondrion and binds MAVS, after which MAVS interacts with TANK binding kinase 1, IKKA, and IKKB^9^. The host cell’s immune and apoptotic response is amplified when MAVS induces phosphorylation and nuclear translocation of IRF3. Reactive oxygen species (ROS), which are mostly generated by mitochondrial oxidative phosphorylation, are potent regulators of MAVS^10^. The direct effects of ROS on MAVS are independent from RNA sensing. ROS promote the formation of the MAVS signaling complex and negatively regulate the expression of MAVS^11^. Hence, mitochondria can modify innate immune signaling either through direct MAVS signaling or through ROS production (e.g., lowering electron transport chain activity, thereby lowering ROS production).

Furthermore, polypeptides encoded by the open reading frames (ORFs) of SARS-CoV-2 have been identified as potential mitochondria interactors. For example, SARS-CoV-2 Orf9b was recently shown to interact with TOM70 and contribute to the largest SARS-CoV-2 proteomic interactome hub^12, 13^. Moreover, SARS-CoV, which was identified in 2002 amid the international SARS outbreak, targeted the mitochondrial-associated adaptor molecule MAVS signalosome^14^. Another adjacent ORF, SARS-CoV Orf9c, is thought to interact with mitochondrial Complex I assembly and mitochondrial ribosome proteins, and SARS-CoV Nonstructural protein 2 (NSP2) has been localized to mitochondrial prohibitin^12, 15^. Further, mitochondrial-gene expression was upregulated in peripheral blood mononuclear cells (PBMC) of infected patients^16^. Therefore, SARS-CoV-2 might affect both the nuclear-encoded mitochondrial transcriptome and mtDNA-encoded gene transcriptome. How SARS-CoV-2 modifies the mitochondrial transcriptome could yield mechanistic directions focused on deciphering innate immune response evasion. In the following analyses, we assessed the effect of SARS-CoV-2 on the mitochondrial transcriptome by reanalyzing publicly available RNASeq data.

## Results

### Selection of COVID datasets

In order to examine the NEM and mtDNA expression signature in SARS-CoV-2 infection, we utilized data sets that were uploaded to GEO (GSE147507 and GSE110551) and the BIG Data Center (CRA002390). These RNASeq data sets were originated from A549, A549 (ACE2), Calu-3, and NHBE cells as well as from SARS-CoV-2 patients’ lung autopsies and bronchoalveolar lavage fluid (BALF). A549 cells were infected with seasonal influenza A virus (IAV), human orthopneumovirus (respiratory syncytial virus; RSV), human parainfluenza virus 3 (HPIV3), and SARS-CoV-2. ACE2-expressing A549 cells, Calu-3 cells, and NHBE cells were infected with SARS-CoV-2. NHBE cells were also infected with IAV. The original authors who curated the in vitro data infected SARS-CoV-2 in A549 cells at low and high multiplicities of infection (MOI). They found that the rate of SARS-CoV-2 replication after low MOI was comparable to the replication rate after high MOI in ACE2-expressing A549 cells^2^. The original authors also observed that low MOI SARS-CoV-2 infection stimulated a relative muted proinflammatory response, which was ablated in high MOI SARS-CoV-2 infection in ACE2-expression A549 cells. We specifically contrasted infection conditions by using low MOI SARS-CoV-2 in order to (1) stay consistent with previously published results and limit confounding effects from stoichiometry disruption of high SARS-CoV-2 components. The number of differentially expressed genes (DEGs) and NEM DEGs per biological source is listed in Table 1. Significant DEGs were filtered by an adjusted *p* value of 0.2.

**Table 1:** The number of differentially expressed genes (DEGs) and NEM DEGs per biological source. A DEG was considered an NEM according to GO:0005739.

Source	Total samples	Total DEGS	Total nuclear-encoded mitochondrial (NEM) DEGs	NEM DEGs as percentage of total DEGs (%)	NEM DEGs as percentage of NEM GO annotations (%)
NHBE	9	2840	313	12.7	17.0
A549 (ACE2)	6	3265	293	9.0	16.1
Calu-3	6	4219	455	10.8	24.7
BALF	5	5353	411	7.7	22.3
Lung	10	475	28	5.9	1.6

### SARS-CoV-2 differentially regulates mtDNA-encoded genes

**Figure 1:**
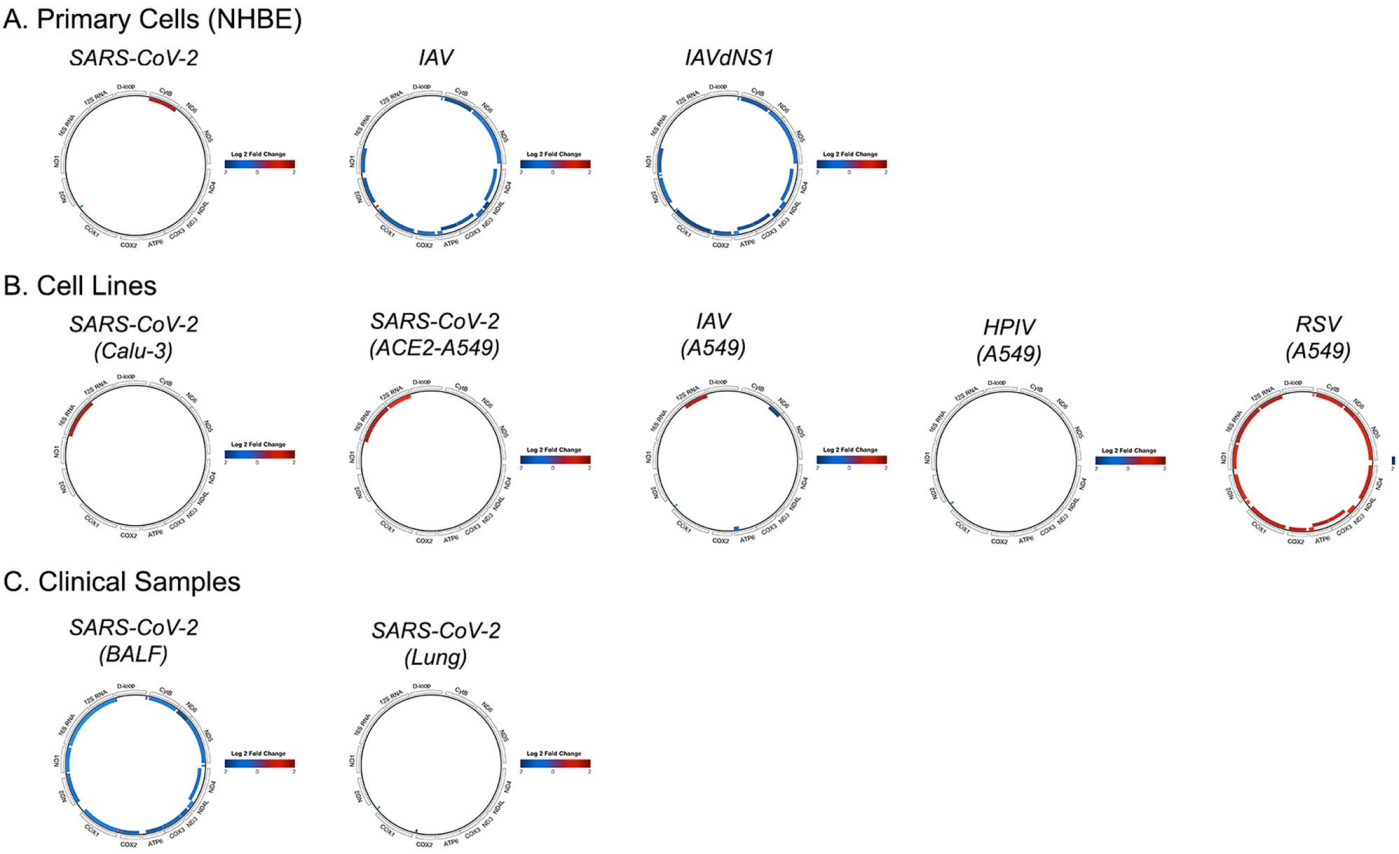
Mitochondrial-gene expression after viral infection in primary cells (**A**), cell lines (**B**), and clinical samples (**C**). Colored genes indicate log twofold change with a padj < 0.2.

We hypothesized that SARS-CoV-2 infection would upregulate mtDNA-encoded gene expression because SARS-CoV upregulated mitochondrial gene expression in patient PBMCs^16^. In our analyses, however, mtDNA-encoded gene expression remained mostly constant. In primary cells, SARS-CoV-2 only increased expression of mt-Cytb, which contrasted the robust mtDNA down regulation of IAV and IAVdNS1 (i.e., IAV with a null interferon antagonist NS1 mutant) (Fig. 1A). In cell lines, SARS-CoV-2 did not upregulate any mtDNA-encoded proteins, but it did upregulate *16S rRNA* in Calu-3 and ACE2-expressing A549 cells (Fig. 1B). RSV, in contrast, upregulated nearly every mtDNA-encoded gene in A549 cells, and HPIV and IAV had minimal effects on mtDNA-ecoded gene expression. Surprisingly, SARS-CoV-2 down regulated nearly every mtDNA-encoded gene along with several mt-tRNAs in BALF (Fig. 1C), an observation that was against our original hypothesis. The complete list of significant mtDNA-encoded genes and fold changes are included in Supplementary Table: mtDNA Differentially Expressed Genes.

### Nuclear-encoded mitochondrial genes sufficiently classifies SARS-CoV-2

**Figure 2:**
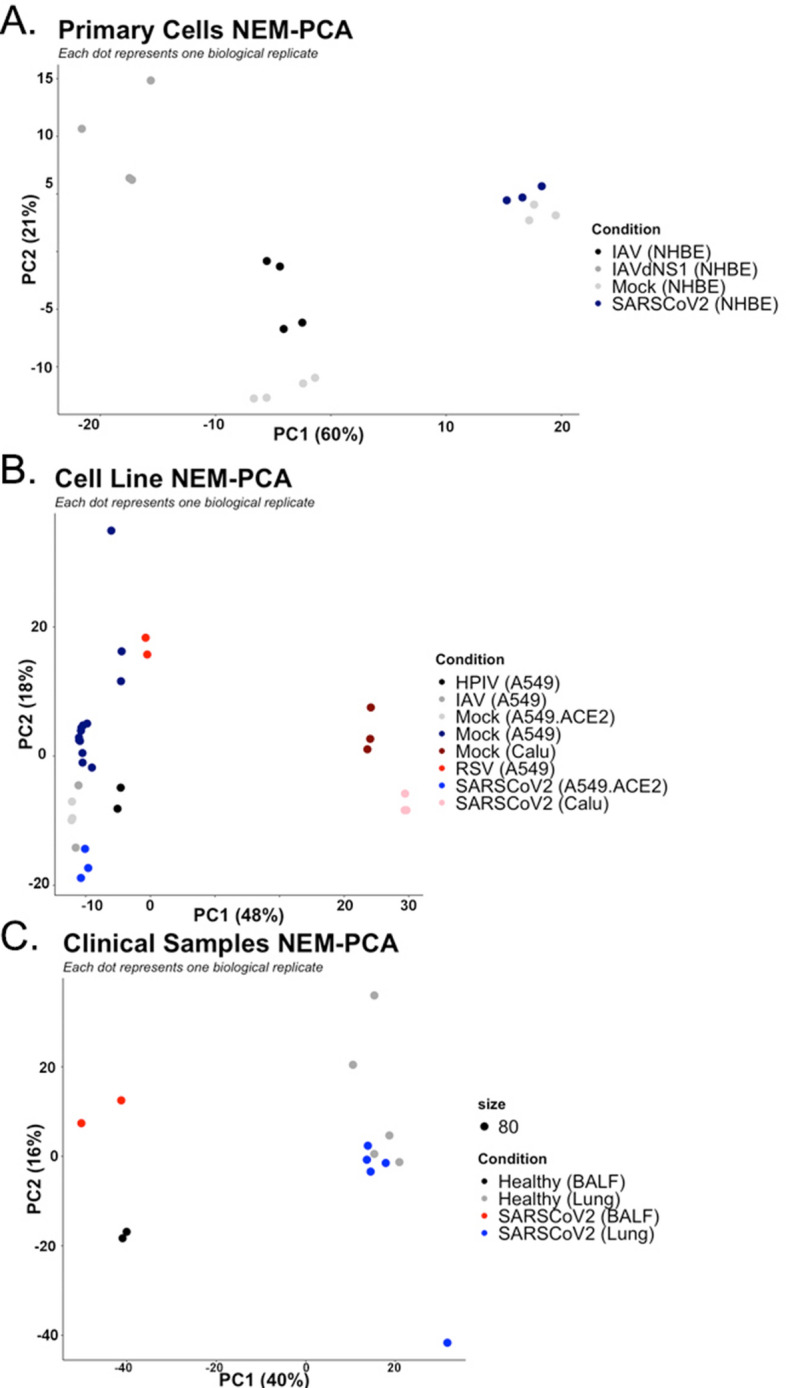
Principal component analysis of NEM expression data by primary cells (**A**), cell lines (**B**), and clinical samples (**C**).

We hypothesized nuclear-encoded mitochondrial genes would sufficiently distinguish SARS-CoV-2. Therefore, we conducted a principal component analysis (PCA) exclusively on an NEM-extracted gene set (i.e., nuclear-encoded mitochondrial genes derived from GO terms). As expected, the first two principal components reduced NEM expression variance in a manner that classified SARS-CoV-2 in primary cells, cell lines, and clinical samples (Fig. 2). The amount of variance that the first two NEM-specific two principal components explain total 81%, 60%, and 56% for primary cells, cell lines, and clinical samples, respectively.

### SARS-CoV-2 enriches pathways related to nuclear-encoded mitochondrial genes

**Figure 3:**
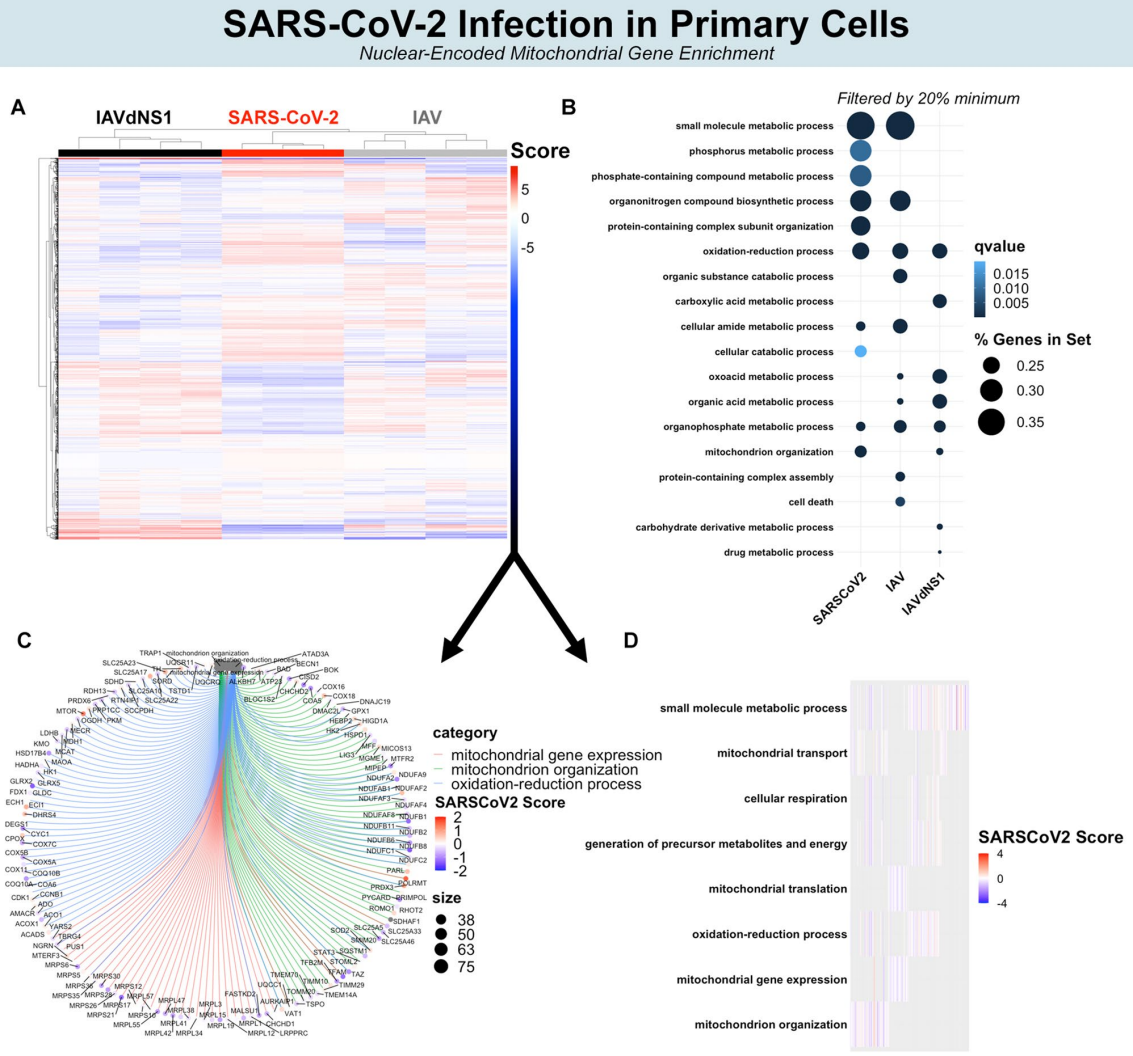
Biological processes affected by NEM expression. Hierarchical clustering of all NEMs separate SARS-CoV-2, IAV, and IAVdNS1 (**A**). Top NEM biological processes by > 20% gene set enrichment (**B**). Top NEM biological processes by q value (**C**). Circos plot illustrating significant NEMs and interconnectedness among biological processes. (**D**) Heat map representing the top 10 GO enrichments.

**Figure 4:**
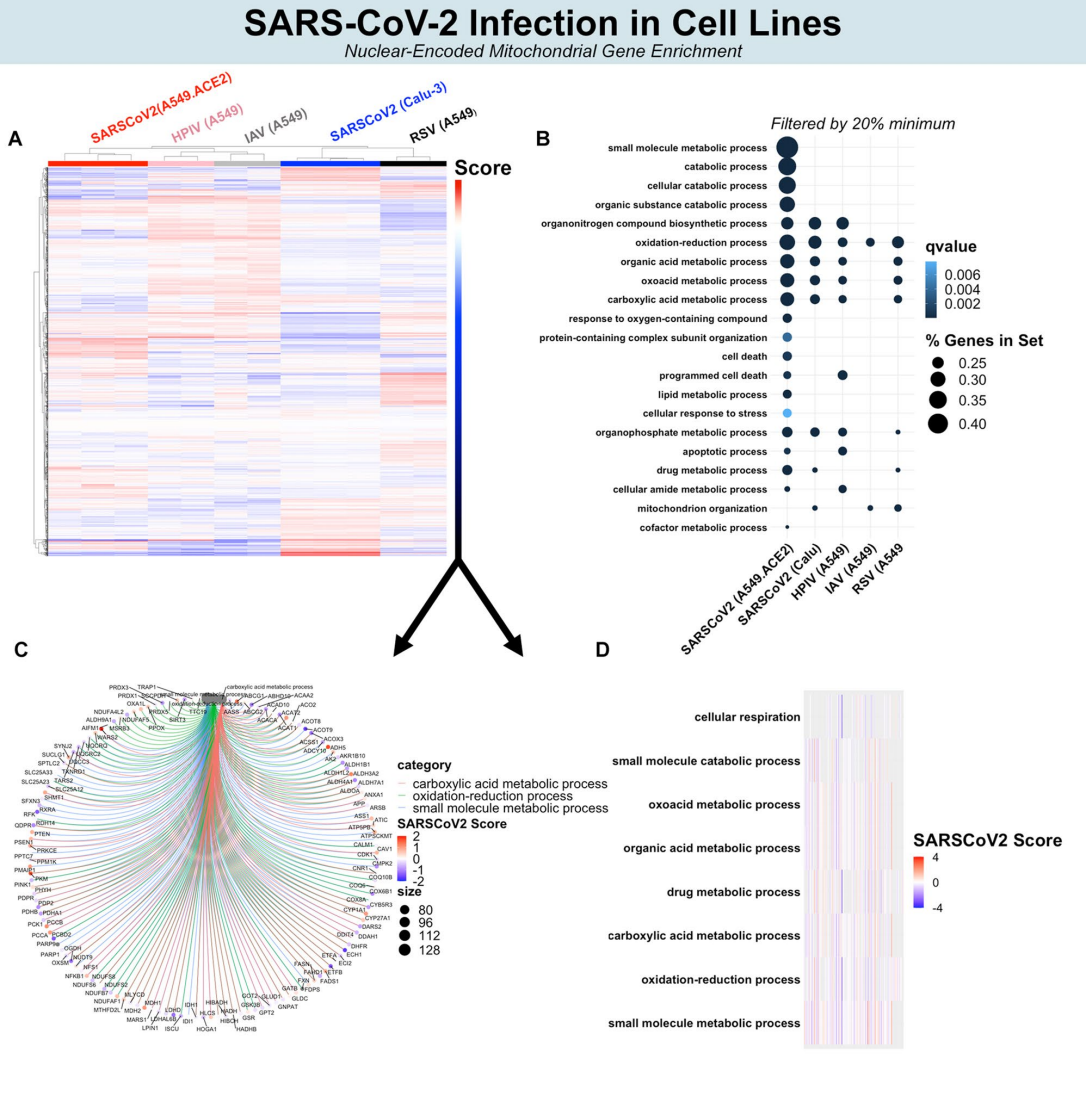
Biological processes affected by NEM expression in cell lines. Hierarchical clustering of all NEMs separate SARS-CoV-2 from other viruses (**A**). Top NEM biological processes by > 20% gene set enrichment (**B**). Top NEM biological processes by q value. (**C**) Circos plot illustrating significant NEMs and interconnectedness among biological processes. (**D**) Heat map representing the top 10 GO enrichments.

**Figure 5:**
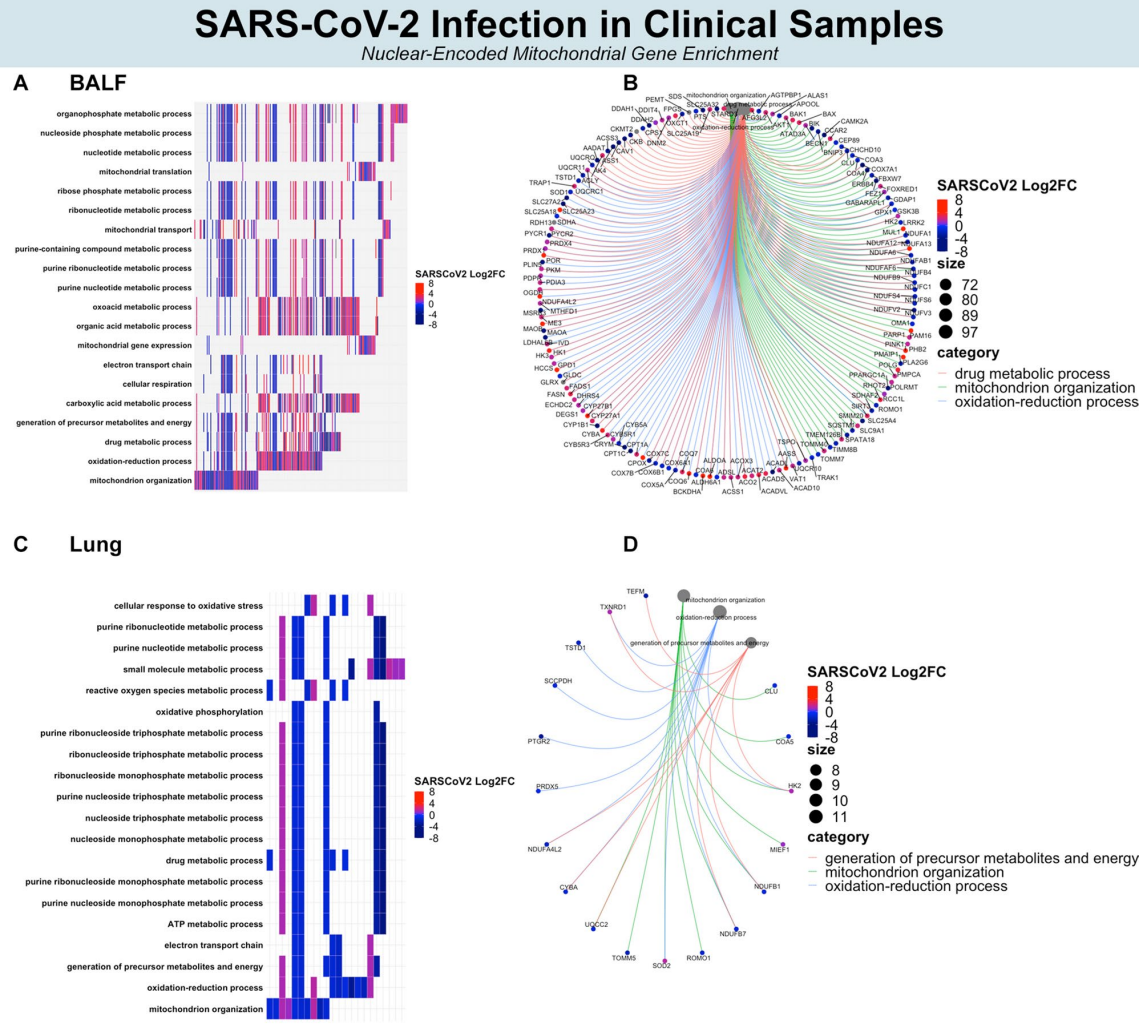
Biological processes affected by NEM expression in clinical. Most significant (by qvalue) NEM-enriched biological processes (**A**, **C**) and corresponding genes in circos plot (**B**, **D**) with color representing log twofold change in BALF and lung, respectively.

Since NEMs classified SARS-CoV-2, HPIV, RSV, and IAV, we attempted to identify the potential functions of these NEMs. Gene enrichment analyses were conducted by inputting this set of significant NEMs against a universe background of all total significant DEGs (i.e., answering the question of, among a set of significant DEGs, which processes are enriched for NEMs). Separate gene enrichment analyses were conducted in primary cells, cell lines, BALF, and lung. Colors in Figs. 3 and 4 represent hierarchical clustering scores of SARS-CoV-2 relative to other viruses (i.e., the colors represent the comparison between SARS-CoV-2 and other viruses). Colors in Fig. 5 represent fold change after SARS-CoV-2 infection.

In primary cells, several mitochondrial ribosome protein genes (e.g., MRPL55, MRPL47, MRPL42, etc.) and Complex I related genes (e.g., NDUFB11, NDUFB2, NDUFC1, etc.) were downregulated and expressed less after SARS-CoV-2 compared to IAV and IAVdNS1 (Fig. 4C,D). The GO enriched terms of small molecule metabolism, phosphorus metabolism, oxidation–reduction, and cellular amide metabolism were all shared between SARS-CoV-2 and IAV in NHBE primary cells (Fig. 3B; filtered by > 20% NEM within gene set). However, SARS-CoV-2 particularly induced greater enrichment for mitochondrion organization and catabolism compared to IAV and IAVdNS1 (Fig. 3B). Furthermore, the top 10 most significant NEM enrichments of SARS-CoV-2 mapped to mitochondrial translation, mitochondrial organization, and cellular respiration (Fig. 3C,D).

In cell lines, mitochondrial Complex I expression (e.g., NDUFS2, NDUFB7, NDUFS6, etc.) also decreased in SARS-CoV-2 compared to IAV, IAVdNS1, HPIV, and RSV (Fig. 4C). As noted in primary cells, enrichment for oxidation–reduction metabolism was shared among all viral infections (i.e., SARS-CoV-2, HPIV, RSV, and IAV). For SARS-CoV-2 in ACE2-expressing A549 cells, a greater number of NEMs were involved in catabolism and small molecule metabolism, although the degree of enrichment was smaller in Calu-3 cells (Fig. 4B). Downregulation of mitochondrial ribosome protein genes was seen in primary cells after SARS-CoV-2 infection, but similar enrichment for mitochondrial translation after SARS-CoV-2 infection was not observed in cell lines. Instead, a greater number of differentially expressed genes related to carboxylic acid metabolism (FASN, ACAT1, ACAT2, etc.) were observed in cell lines. The top 10 most significant enriched processes after SARS-CoV-2 in ACE2-expressing A549 cells mapped to cellular respiration, oxidation–reduction, small molecule metabolism, among many other interrelated metabolic pathways (Fig. 4C). Overall, attenuation of cellular respiration (mitochondrial Complex I genes), oxidation–reduction, and related interconnected pathways occurred in cell lines after SARS-CoV-2 infection relative to IAV, HPIV, and RSV.

SARS-CoV-2 BALF and lung clinical samples also contained decreased expression of genes involved in cellular respiration and Complex I (BALF: NDUFAF6, NDUFB9, NDUFV2, etc.; Lung: NDUFB1, NDUFB7, NDUFAL2). However, the effect of SARS-CoV-2 on the NEM transcriptome was not as dramatic in lung compared to BALF. In BALF, the most significant NEM-enriched sets included organophosphate metabolism, mitochondrial gene expression, cellular respiration, oxidation–reduction, etc. (Fig. 5A,B). The majority of these genes were also downregulated in SARS-CoV-2 infected primary cells and cell lines. In lung, despite identifying just 28 significant NEMs, mitochondrion organization, phosphorus metabolism, and overall energy metabolism were enriched (Fig. 5C,D).

Here, the SARS-CoV-2-specific signature included decreased expression of NEMs involved in cellular respiration and Complex I assembly (NDUFs) across all models. Mitochondrial ribosome gene expression was particularly downregulated after SARS-CoV-2 infection in primary cells (greater downregulation compared to IAV) and clinical samples. There were clear tissue and cell-specific differences across all analyses related to oxidation–reduction, small molecule metabolism, and carboxylic metabolism, suggesting SARS-CoV-2 may affect metabolism differently per cell type or per experimental condition that must be considered as potential therapeutics continue to be tested. Nevertheless, that Complex I expression was lower in SARS-CoV-2 infected cells compared to other respiratory viral infected cells suggests a mechanism for innate immunity evasion.

### SARS-CoV-2 does not change MAVS expression

**Figure 6:**
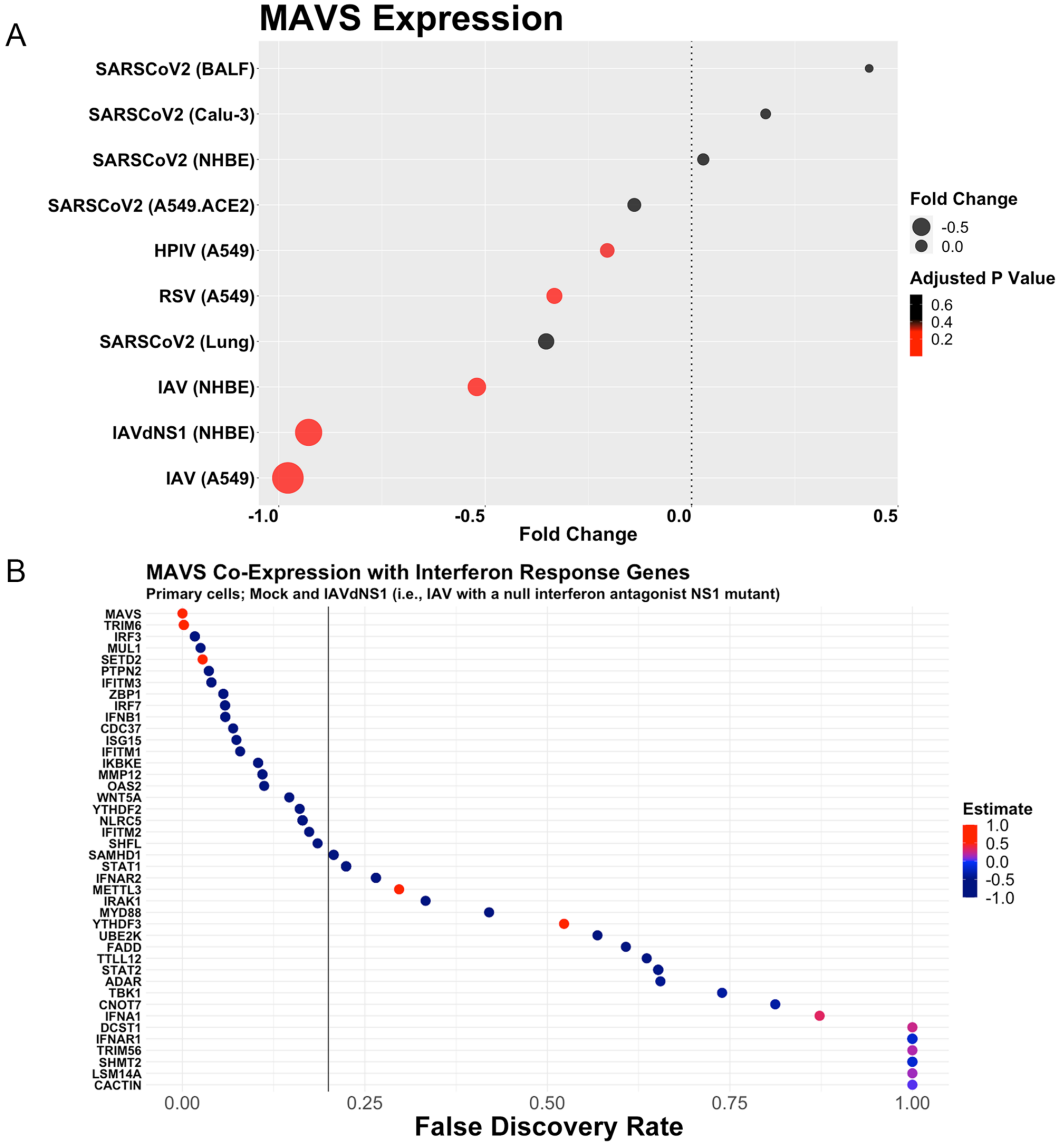
SARS-CoV-2 does not induce downregulation of MAVS, whereas HPIV, RSV, and IAV downregulate MAVS across cell types (**A**). MAVS expression is inversely correlated with interferon response gene (**B**).

We hypothesized that MAVS expression would not change after SARS-CoV-2 infection due to prior reports on SARS-CoV inhibiting MAVS. Indeed, we did not observe significant MAVS expression changes for SARS-CoV-2 in ACE2-expressing A549 cells, Calu-3 cells, NHBE cells, BALF, and lung (Fig. 6A). In contrast, IAV, RSV, and HPIV all induced a statistically significant downregulation of MAVS. IAV-infected A549 cells induced the most dramatic downregulation of MAVS (Log2FC = − 0.98; Padj = 1.32E−08), followed by IAVdNS1 in NHBE cells (Log2FC = − 0.93; Padj = 5.35E−04), IAV in NHBE cells (Log2FC = − 0.52; Padj = 1.11E−01), RSV in A549 cells (Log2FC = − 0.33; Padj = 7.00E−02) and HPIV in A549 cells (Log2FC = − 0.20; Padj = 2.02E−01).

Moreover, we conducted a co-expression analysis for MAVS and every interferon response gene (as annotated by GO Ontology). The co-expression analysis was conducted in cells infected with mock and IAVdNS1 due to the null interferon antagonist NS1 mutant. We found that MAVS expression inversely correlated with the majority of interferon-response genes (Fig. 6B). That is, higher MAVS expression correlated with a lower interferon response. Our finding that SARS-CoV-2 did not affect MAVS expression aligns with a previous report describing an imbalanced SARS-CoV-2 innate immune transcriptomic response^2^.

## Discussion

We concentrated on the mitochondrial and nuclear-encoded mitochondrial gene host response to SARS-CoV-2 and other human respiratory viruses in multiple cell models and clinical samples. Our analyses showed that the mitochondrial transcriptome signature of SARS-CoV-2 infection included both shared and independent biological processes from IAV, HPIV, and RSV. We observed three mitochondrial-related transcriptomic signature differences: (1) mtDNA-encoded gene expression, (2) nuclear-encoded mitochondrial gene enrichment, and (3) MAVS expression.

We found that SARS-CoV-2 did not induce a dramatic mtDNA-encoded gene expression signature in NHBE, A549, and Calu-3 cells. This contrasts the downregulatory effects of IAV and IAVdNSA1 on mtDNA-encoded gene expression in NHBE cells. We propose three explanations for the minimal effects of SARS-CoV-2 on mtDNA-encoded gene expression. First, SARS-CoV-2 Orf9b localized to TOM70 on the mitochondrion^13^. Under interferon activation conditions, HSP90 binds TOM70^17^. However, since Orf9b also binds TOM70, it is possible that the HSP90/TOM70 complex is inhibited; therefore, the mitochondrial cellular environment might be similar to resting conditions. TOM70 is essential for mitochondrial oxidative phosphorylation energy production and the import of nuclear-encoded transcription factor A (TFAM)^18^. Second, the effects of SARS-CoV-2 on mtDNA-encoded gene expression might not be exclusive to electron transport chain protein production. We observed upregulation of POLRMT, a gene involved in catalyzing the transcription of mtDNA-encoded genes^19^. Given that we observed greater expression of POLRMT but not mtDNA-encoded gene expression, perhaps SARS-CoV-2 modifies the expression of mtDNA with non-protein-encoding functions. That is, perhaps SARS-CoV-2 acts on the mitochondrion to modulate non-protein coding functions of mitochondrial RNA that our analyses did capture. The mitochondrial transcriptome is highly complex with dozens of cleavage sites that yield mitochondrial RNA species with unknown function^20^. These mitochondrial RNA species could be non-protein coding or part of a system that encodes for small open reading frame mitochondrial-derived peptides (MDPs)^21–23^. Third, considering we did not observe upregulation of nuclear-encoded genes that are part of cellular respiration after SARS-CoV-2 infection, it is possible that mtDNA-encoded gene expression remained similar to that at resting condition levels. Unperturbed mitochondrial transcription might promote a stable intracellular ROS environment. Additional explanations will undoubtedly be proposed as more data is curated on SARS-CoV-2^23–25^.

In clinical samples, we observed dramatic downregulation of mitochondrial-gene expression in human BALF after SARS-CoV-2 infection, although we did not observe the same degree of downregulation in human lung. The cellular profile of BALF after infection differs significantly compared to resting state conditions. Prior reports have shown nearly a 900-fold increase in neutrophil infiltration after LPS stimulation^26^. Hence, it is possible that decrease in mtDNA-encoded gene expression that we observed in SARS-CoV-2 patient BALF is a byproduct of a different cellular profile, and it is also possible that SARS-CoV-2 affects mitochondria in immune cells differently than in lung cells.

Furthermore, we observed that, across multiple cell and tissue types, SARS-CoV-2 reduced NEM expression related to cellular respiration and Complex I. We found many NDUF proteins were downregulated after SARS-CoV-2 infection in NHBE cells, cell lines, BALF, and lung. Complex I is one of the main contributors to ROS production. It is plausible that muted expression of Complex I—and therefore lower ROS production—permits SARS-CoV-2 propagation. Additional reports on respiratory viruses such as RSV have indicated that Complex I inhibition could promote efficient viral replication^27^. SARS-CoV-2 not only decreased expression to many NDUF family of genes, but also several mitochondrial ribosome protein related genes in primary cells and BALF, observations of which, however, did not carry over to cell lines. It is possible that the cancerous nature of A549 and Calu-3 cells limits our interpretation on metabolism due to naturally different cellular metabolic features^28^. Nevertheless, SARS-CoV-2 ORF9c has been previously reported to interact with mitochondrial NDUFAF1, NDUFB9, MRPS2, MRPS5, MRPS25, and MRPS27; the direct interaction between SARS-CoV-2 and these Complex I and mitochondrial ribosome proteins could explain why we observed transcriptomic downregulation of such processes after SARS-CoV-2 infection^12^.

In addition, we found that SARS-CoV-2 did not alter MAVS expression, whereas IAV, HPIV, and RSV all decreased MAVS expression. We also reported that MAVS expression is lower when interferon-response transcription is activated. Considering that IAV, HPIV and RSV all decreased MAVS expression and induced a greater interferon response, it is possible that MAVS is a direct target by SARS-CoV-2. Perhaps the binding of SARS-CoV-2 Orf9b with TOM70 (which binds MAVS) competes with MAVS binding, thereby sustaining cytoplasmic MAVS levels and thus not affecting regulation of MAVS^13^. Since expression genes related to Complex I were lower in SARS-CoV-2 infected cells, it is also conceivable that MAVS expression was lower due to lower ROS production.

Model heterogeneity related to cellular catabolism, lipid processing, and carboxylic acid metabolism was observed in our analyses. We noted a discordant amount of NEMs related to lipid processing and carboxylic acid metabolism across cell and tissues after SARS-CoV-2 infection. For example, we observed a greater share of NEMs related to carboxylic acid in primary cells after SARS-CoV-2 infection relative to other viruses, while we observed greater shares after other respiratory viral infections in cell lines. This suggests future research should carefully consider the in vitro model, especially if the desired outcome of interest is closely related to mitochondrial biology.

Overall, SARS-CoV-2 did not potently regulate mtDNA-encoded gene expression or MAVS expression and prompted lower expression of Complex I genes compared to other common respiratory viruses. These results complement recent publications highlighting biophysical interactions between mitochondrial proteins and SARS-CoV-2 proteins. Published reports have characterized SARS-CoV-2 Orf9b as a TOM70 interactor, and the Orf9c has been shown to bind proteins involved in Complex Iand mitochondrial ribosome complexes^12^. Another recent report predicted SARS-CoV-2 RNA localization to the mitochondrion^29^. Perhaps the localization of SARS-CoV-2 RNA anneals with mitochondrial deubuiquitanse USP30, a subunit of ubuquiting protein ligase complex FBX021 (https://www.biorxiv.org/content/10.1101/2020.04.08.031856v3.full). SARS-CoV-2 RNA acting as an RNAi might explain many of the downregulatory expression effects that we observed. Future work might consider mitochondrial biology as a primary target for SARS-CoV-2. Our analyses may be used to propose targeted hypotheses that can be addressed in vitro and in vivo.

## Methods

The molecular preparation workflow for the RNASeq data used here has been reported previously^2^. We downloaded sample FASTQ files from GEO or BIG Data Center. These FASTQ reads were mapped to rRNA sequences in order to remove cytoplasmic rRNA reads using STAR (v2.7.2b) with default parameters. Bioinformatically-filtered cytoplasmic rRNA FASTQ files were then mapped to the human genome (hg38) with GENCODE gene annotation (v33) using the following STAR parameters: sjdbScore 1, outFilterMultimapNmax 20, outFilterMismatchNmax 999, outFilterMismatchNoverReadLmax 0.04, alignIntronMin 20, alignIntronMax 1000000, alignMatesGapMax 1000000, and alignSJoverhangMin, alignSJDBoverhangMin 1. Aligned sorted BAM files were inputted into RR (v3.5.1) for counting. A count matrix and corresponding metadata were sorted in an S4 class derived from the *SummarizedExperiment* class of the *GenomicRanges* package in R. Count matrices were generated using the *summarizezeOverlaps* function of the *GenomicAlignemnts* package in R. Overlapping genomic features were resolved using the “Union” mode in the *summarizeOverlaps* function. Differential expression analyses were conducted using the *DESeq* R package. The dds object was transformed by variance stabilization using the *varianceStabilizingTrasnformation* function, which was used during the principal component analysis (*plotPCA* function in R) and euclidean heirarchial clustering (*pheatmap* function in R) scaled to each condition. Significant differentially expressed genes were filtered by a padjusted value of 0.2. Genes under the “mitochondrion” Gene Ontology (GO) Term (GO:0005739) were downloaded and used for NEM enrichment analyses (1842 NEMs). This NEM gene set was used to filter genes from the dds object. Genes under the “response to type I interferon” GO Term (GO:0034340) was extracted for the MAVS co-expression analysis. The MAVS co-expression analysis was conducted via Spearman correlation on normalized counts. Mitochondrial DNA expression heat maps were built using custom scripts in R. NEM enrichment was conducted using the *clusterProfiler* package in R. For GO analyses, the NEM-extracted gene set from statistically significant DEGs were tested against a universe background gene set of all statistically significant DEGs. This approach was implemented in order to identify biological processes that NEMS enriched compared to the background of all DEGs. The cut-off criteria for GO analysis were *p* < 0.05 and q < 0.20. Significant enriched terms were visualized using the two *clusterProfiler* package functions *heatplot* and *cnetplot*.

## Supplementary information

Supplementary Information.
